# Segregation of Regulatory Polymorphisms with Effects on the Gluteus Medius Transcriptome in a Purebred Pig Population

**DOI:** 10.1371/journal.pone.0035583

**Published:** 2012-04-24

**Authors:** Angela Cánovas, Ramona N. Pena, David Gallardo, Oscar Ramírez, Marcel Amills, Raquel Quintanilla

**Affiliations:** 1 IRTA, Genètica i Millora Animal, Lleida, Spain; 2 Departament de Ciència Animal i dels Aliments, Facultat de Veterinària, Universitat Autònoma de Barcelona, Bellaterra, Barcelona, Spain; 3 Departament de Genètica Animal, Centre de Recerca en Agrigenòmica (CRAG), Universitat Autònoma de Barcelona, Bellaterra, Barcelona, Spain; University of Queensland, Australia

## Abstract

**Background:**

The main goal of the present study was to analyse the genetic architecture of mRNA expression in muscle, a tissue with an outmost economic importance for pig breeders. Previous studies have used F_2_ crosses to detect porcine expression QTL (eQTL), so they contributed with data that mostly represents the between-breed component of eQTL variation. Herewith, we have analysed eQTL segregation in an outbred Duroc population using two groups of animals with divergent fatness profiles. This approach is particularly suitable to analyse the within-breed component of eQTL variation, with a special emphasis on loci involved in lipid metabolism.

**Methodology/Principal Findings:**

*GeneChip Porcine Genome* arrays *(Affymetrix)* were used to determine the mRNA expression levels of *gluteus medius* samples from 105 Duroc barrows. A whole-genome eQTL scan was carried out with a panel of 116 microsatellites. Results allowed us to detect 613 genome-wide significant eQTL unevenly distributed across the pig genome. A clear predominance of *trans-* over *cis-eQTL*, was observed. Moreover, 11 *trans-*regulatory hotspots affecting the expression levels of four to 16 genes were identified. A Gene Ontology study showed that regulatory polymorphisms affected the expression of muscle development and lipid metabolism genes. A number of positional concordances between eQTL and lipid trait QTL were also found, whereas limited evidence of a linear relationship between muscle fat deposition and mRNA levels of eQTL regulated genes was obtained.

**Conclusions/Significance:**

Our data provide substantial evidence that there is a remarkable amount of within-breed genetic variation affecting muscle mRNA expression. Most of this variation acts in *trans* and influences biological processes related with muscle development, lipid deposition and energy balance. The identification of the underlying causal mutations and the ascertainment of their effects on phenotypes would allow gaining a fundamental perspective about how complex traits are built at the molecular level.

## Introduction

Expression quantitative trait loci (eQTL) mapping represents a valuable approach towards identifying regulatory regions and DNA sequence variants affecting the expression levels of genes. Genetical genomics studies performed in human have indicated that: (*i*) eQTL maps obtained in different cell types are partially concordant [1], [2], *i.e.* often regulatory polymorphisms exert their effects on multiple tissues, and (*ii*) these maps might differ amongst populations with different genetic backgrounds [3], [4], mainly because of differences in the frequency of regulatory alleles rather than in the specific set of mechanisms involved in the fine-tuning of mRNA levels [5]. More controversy exists about the abundance of regulatory hotspots in the human genome, which have been identified in certain studies but not in others, and the relative impact of *cis-*eQTL *vs trans-*eQTL on transcriptome variation [5]. In general, studies performed in model organisms have been more successful at identifying regulatory hotspots and *trans-*eQTL than those performed in human, probably because of experimental rather than biological reasons [5], [6].

So far, the few eQTL studies performed in pigs [7], [8], [9], [10] are based on the analysis of samples obtained from crossbred individuals (*e.g.* Duroc x Pietrain and Large White x Landrace). This experimental design is particularly suited to capture the between-breed component of genetic variation. Although useful, eQTL maps obtained in crossbreds do not provide an accurate account of the regulatory DNA variation segregating in purebred swine populations, where intensive selection, inbreeding, founder effects and genetic drift have strongly modified genetic diversity.

In the current work, we have analysed the segregation of muscle eQTL in two groups of Duroc pigs with divergent phenotypes for fat deposition traits. Important differences in the expression of cell differentiation, energy balance and fat metabolism genes between these groups had been detected in a previous study [11]. Our main goal was to dissect the within-breed component of eQTL variation in a purebred population with a special focus on the regulation of lipid metabolism- and fatness-related genes.

## Results and Discussion

### Segregation of expression Quantitative Trait Loci regulating gluteus medius mRNA levels in a commercial Duroc pig population

**Figure 1:**
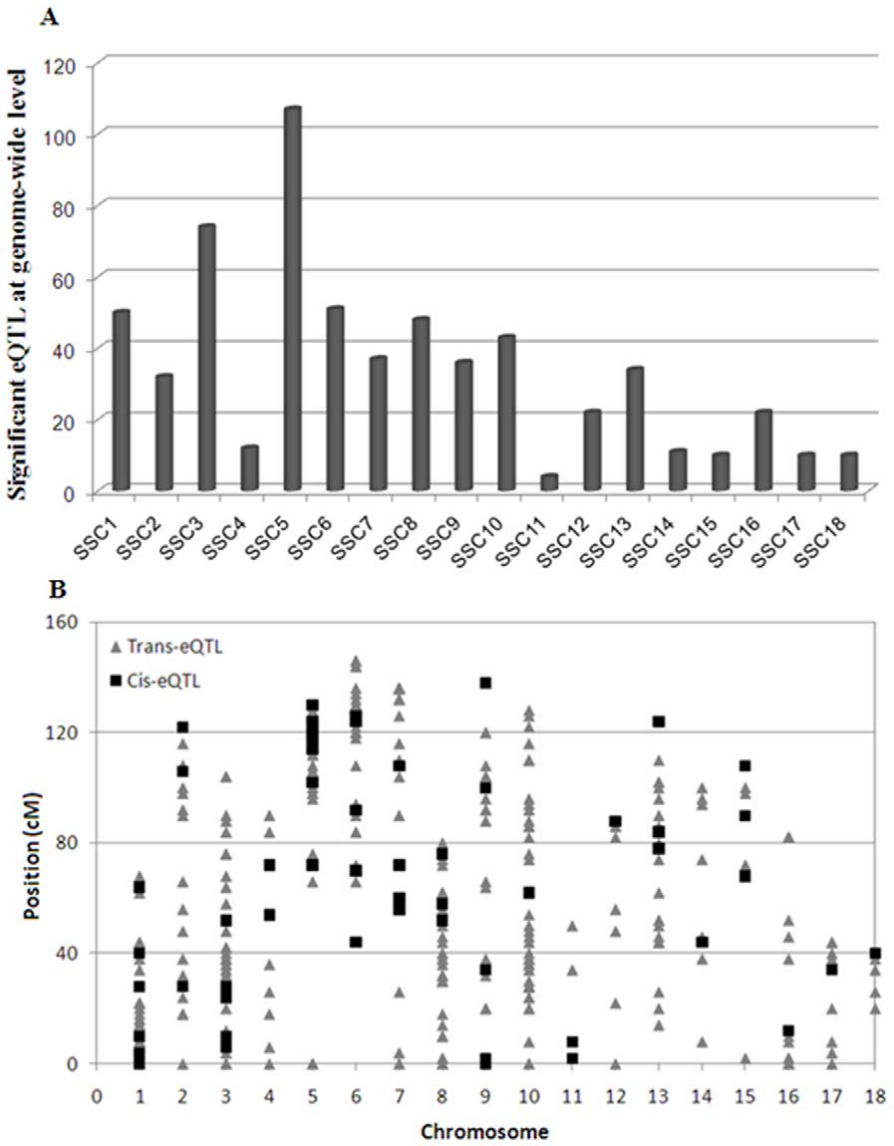
Distribution of genome-wide expression QTL amongst pig chromosomes. (A) Total number of eQTL and (B) *cis-*acting (▪) and *trans-*acting (▴) eQTL.

In the whole-genome scan carried out for 6,139 *trans*cripts, a total of 613 genome-wide significant eQTL (*P*-value cut off *P*<0.0009) affecting the mRNA expression of 569 probes were identified (Table 1). There was an uneven distribution of these eQTL across the 18 autosomes (Figure 1), with *Sus Scrofa Chromosomes* (SSC) 3 and 5 harbouring the highest number of eQTL (74 and 107 respectively). In contrast, only three and seven eQTL were observed on SSC11 and SSC14, respectively. Out of the 569 total eQTL-targeted probes, 490 were successfully mapped to the pig genome (Table 1), although a small fraction (12) corresponded to non-specifically mapped probes. The high number of unmapped probes (123 probes; 20%) is due to the incomplete annotation of the porcine genome sequence currently available.

**Table 1:** Distribution of high and low quality porcine *Affymetrix* probes (and corresponding number of genes) regulated by either *cis-* or *trans-*acting eQTL.

	N. eQTL, N. affected probes, N. genes
Total	613 eQTL, 569 probes, 536 genes
eQTL with mapped target-probes	478 eQTL, 451 probes, 427 genes

	*cis-* acting eQTL	*trans-* acting eQTL
**Distribution of ** ***cis-*** ** and ** ***trans-*** **eQTL:**	63 eQTL, 63 probes, 62 genes	415 eQTL, 393 probes, 374 genes
**Low quality target probes**		
23 eQTL, 21 probes, 21 genes	4 eQTL, 4 probes, 4 genes	19 eQTL, 18 probes, 18 genes
**High quality target probes**		
455 eQTL , 430 probes, 407 genes	59 eQTL, 59 probes, 58 genes	396 eQTL, 377 probes, 357 genes

The relative position of the eQTL and the target genes was studied in order to classify them into *cis-*acting and *trans-*acting eQTL. From the 478 mapped eQTL, only 63 were catalogued as *cis-*acting eQTL on the basis of their close physical distance to the target gene, whereas the remaining 415 eQTL were considered to regulate gene expression in *trans* (Table 1). Finally, only eQTL whose target probes showed high quality binding of the probe-sets (*i.e.* six or more out of 11 probes specifically assembled to a unique region) were retained for further analysis (59 *cis-* and 396 *trans-*eQTL; Table 1). Whole data regarding these 455 eQTL (eQTL peaks, target gene locations, flanking markers, confidence intervals, distances from the closest marker and classification as cis-/trans- eQTL) is provided in Table S1.

Strong evidences of muscle eQTL segregation had been previously reported by Steibel et al. [9] in a population of 176 F_2_ Duroc x Pietrain and by Liaubet et al. [10] in 57 F_2_ Pietrain x composite line (Hampshire, Duroc and Large White) pigs, but no studies on the segregation of eQTL in porcine purebred population have been carried out so far. As a whole, the number and magnitude of muscle eQTL detected in the present study were comparable to those reported by Steibel et al. [9], who described 397 putative eQTL peaks using a first *P*-value cut off (*P*<0.0001), which is slightly more conservative than ours (*P*<0.0009). Similarly, Liaubet et al. [10] described 335 eQTL significant at a chromosome-wide empirical threshold of 1%. Given the high cut-off values applied to correct for multiple testing, the hundreds of eQTL identified in our and other studies is expected to be the tip of the iceberg (*i.e.* many small-effect alleles are missed because of a lack of statistical power to detect them). This is particularly meaningful in a purebred population such as the one described in the current work, where allelic frequencies could have been substantially modified by selection, inbreeding and genetic drift.

Although genetic variability of our Duroc population should be much lower than that detected in F_2_ crosses, it is relevant to highlight that in previous studies we detected moderate to high heritability estimates [12] and a wide number of QTL for several lipid metabolism and fat deposition traits [13], [14], [15]. Probably, both the inbreeding control programme and the simultaneous selection for multiple breeding objectives diminished the impact of selection, in terms of reducing variability. Moreover, the half-sibs family design employed in the current work (five boars mated to minimally related females) allowed us minimizing inbreeding in the analysed population (F = 0.006; computed from a pedigree of 1630 ancestors). These circumstances have facilitated the identification of a remarkable level of regulatory allelic variation at the within-breed level, even comparable with that detected in F_2_ divergent crosses [9], [10]. The current findings imply that selecting beneficial gene expression profiles, in terms of carcass or meat quality, would be feasible from a theoretical point of view.

### A majority of trans-acting eQTL modulate gene expression in the gluteus medius muscle of Duroc pigs

We have detected a much higher percentage of *trans-*acting than *cis-*acting eQTL (396 *vs* 59 respectively) in the pig muscle. Following Ponsuksili et al. [16], we considered eQTL as *cis*-acting when both the eQTL peak and the target gene fell within the interval delimited by the two flanking markers of the eQTL (Table S1). It is worth noting that our map resulted in relatively large confidence intervals for eQTL positions (average 31.40 cM), while the average distance from the closest marker was 7.39 cM. These relatively large confidence intervals lead to classify as *cis-*acting most eQTL that happen to be located in the same chromosome that the corresponding target gene. Thus, it must be taken into account that some *trans-*eQTL could have been misclassified as *cis-*eQTL.

**Figure 2:**
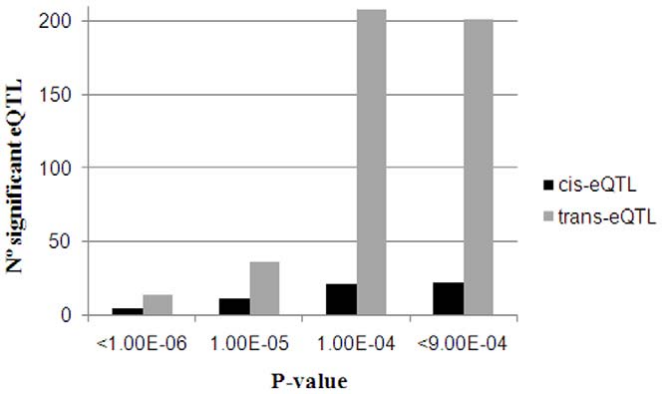
Effect of the genome-wide *P*-value cutoff on the relative frequencies of *trans*- and *cis*-eQTL.

The relative importance of *cis-*eQTL *vs trans-*eQTL is still under debate, with the former predominating in certain studies [2], [17] and the latter in other ones [16], [18], [19]. Since basic regulatory mechanisms amongst mammals are not expected to differ substantially, either technical or experimental factors might probably be the main source of these discrepancies. There are several reasons by which *cis*-eQTL should be more frequently identified in whole-genome scans than *trans-*eQTL. First of all, and according to Cheung et al. [5], *trans-*eQTL might have smaller effects on individual genes than *cis-*eQTL (*i.e.* genes are regulated by many *trans-*acting factors but by a few *cis-*acting factors), so they would be more difficult to detect. Moreover, multiple-testing thresholds are stricter for *trans-*eQTL than for *cis-*eQTL [6]. In the line of these arguments, Steibel et al. [9] highlighted that local (*cis-*acting) eQTL were in general much more significant (smaller *P*-values) than *trans-*acting eQTL. In summary, given the conservative thresholds applied to correct for multiple testing and the smaller effects expected for the *trans-*eQTL, a high number of *trans-*regulatory polymorphisms might be undetected in studies burdened by low sample size and/or less powerful designs. However, and in strong contrast with this theoretical expectation, our study and others [16], [18], [19] have highlighted a higher abundance of *trans- vs cis-*eQTL. Importantly, this predominance of *trans-*eQTL decreases substantially when more stringent *P-*value cutoffs are employed to assess the significance of the data. For instance, when Steibel et al. [9] applied a multiple-testing correction with a *P*-value of 3.5×10^−6^ (corresponding to a FDR of 10%), 40 out of 62 significant eQTL had linkage peaks on the same chromosome where the regulated probe was physically located. Results obtained in the current study were also consistent with this statement: the predominance of *trans-* over *cis-*eQTL decreased dramatically as the significance threshold increased (Figure 2), although in our case *trans-*acting eQTL were more frequent than the *cis-*ones even with the most conservative *P*-value cut-off (*P*<10^−6^). Another plausible hypothesis that would be worth to investigate is whether a relatively lower genetic variation, as expected in our outbred population subjected to selection, could result in gene regulatory networks (*trans*-eQTL) standing out when compared to gene regulatory elements (*cis*-eQTL). In any case, the higher frequency of *trans*-eQTL probably reflects a biological reality since, as discussed above, experimental factors usually lead to underestimate the number of *trans*-eQTL. In this sense, Cheung et al. [20] demonstrated that when large datasets are used, *trans*-eQTL are also more abundant than the *cis*-ones in human.

### The pig genome contains regulatory hotspots with effects on the muscle transcriptome

**Figure 3:**
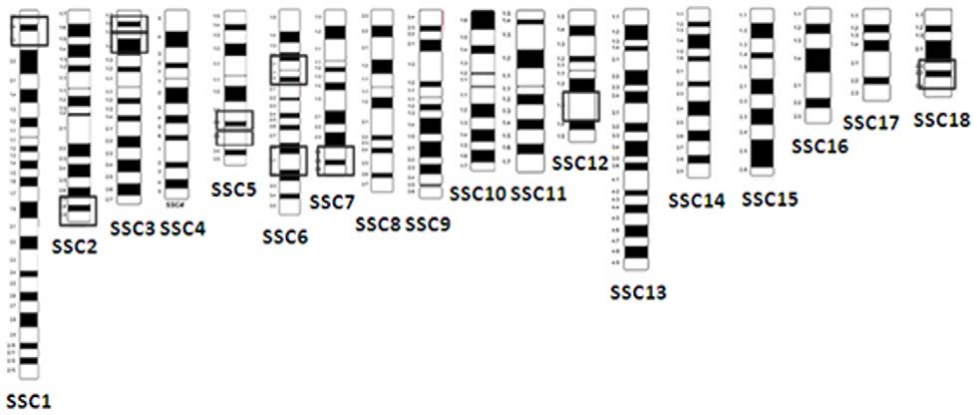
Porcine genomic regions with eQTL regulating more than four genes (eQTL hotspots).

In principle, *trans-*eQTL are expected to affect the expression of many genes while *cis-*eQTL must (necessarily) have a more restricted effect. A detailed analysis of eQTL affecting the expression level of several genes led us to define 11 eQTL hotspots (Figure 3), mapping to SSC1, SSC2, SSC3, SSC5, SSC6, SSC7, SSC12 and SSC18, and regulating the expression levels of four to 16 genes (Table 2). In close resemblance with our results, Liaubet et al. [10] found that 50% of muscle eQTL clustered to six pig chromosomes. For instance, they detected a SSC1 region influencing the expression of 31 genes, 26 of which displayed a pattern that was consistent with co-regulation by a common genetic determinant. Also Steibel et al. [9] reported 16 peaks on pig genome regions *trans-*regulating more than four genes, despite posterior multiple-testing correction led to discard an important number of *trans-eQTL* and to conclude that *trans-*regulation hotspots are, in general, scarce.

**Table 2:** List of genes regulated by each one of the 11 *trans-*regulatory hotspots regions.

Chromosome	Position	N regulated genes	List of regulated genes
**SSC1**	10 cM	11	*ARPC1A, CBPG, CKS2, CLDND1, CXXC5, G3BP1, IGFBP5, MYO6, RBM25, THOC1, WDR70*
**SSC2**	122 cM	7	*ACOT6, EHD1, UFD1L, SEC22B, FUCA1, ZNF236, C10orf28*
**SSC3**	24 cM	16	*PPP1CB, LCMT1, MEPCE, WASL, CPEB4, TANK, EDEM3, RRM2B, RDX, KCNMB3, KDELR2, THUMPD1, CASD1, PLOD1, TRPM7, EMSP1*
	30 cM	14	*EHD1, IGF1, TRIB2, NNAT, VCPIP1, BDKRB1, GUSB, RBM39, SAP30, GATAD2A, DUSP7, EBAG9, EMD, FAM20C*
**SSC5**	114 cM	16	*PRKAG2, TRPM7, LYRM2, ALDH1L2, EIF3A, MCFD2, HNRNPH1, PRKAA2, HIGD1A, AGRP, ISOC2, PSMC6, PRPF39, CYCS, THUMPD1*
	124 cM	13	*SLC5A1, TMEM44, CUL1, NGAP, APOBEC1, NRXN1, HSD11B1, SF3B1, HMGB2, HRAS, MRPS18C, PFMK, TAF1D*
**SSC6**	66 cM	10	*LRP6, PAFAH1B3, GARS, KIAA1217, DR1, MTSS1, ARF3, LOC654323, SIGLEC10, TM2D2*
	124 cM	9	*CEBPG, HRC, FST, MED10, TRIM44, RBM12B, HDGF2, SS18, CAST*
**SSC7**	134 cM	14	*ACACA, FABP5, CKAP4, PDE7A, NADK, BAG3, PUS1, ACLY, SLC5A6, ZAN, CNKSR3, CLDN9, GPR160, PTPLB*
**SSC12**	88 cM	13	*TAOK1, COL1A1, TMEM98, TRIP11, IGFBP6, PAFAH1B3, C20orf79, OBFC1, CCDC39, TRIM27, FAM98C, CUX1, IL18BP*
**SSC18**	40 cM	4	*CYP24A1, DNAJB6, HOXA10, TPPP3*

Studies in other organisms have identified hotspots containing genetic variants that influence multiple gene expression phenotypes [21], [22]. Expression QTL hotspots have also been reported in the human genome [2], [18]. As pointed out by Morley et al. [18], mRNAs regulated by a common genetic hotspot often share similar functions and are co-expressed. For instance, we identified a *trans-*regulatory hotspot at SSC7 that agreed well with this principle, as it regulated genes with crucial roles on lipid metabolism (*e.g.ACACA*, *ACLY* or *FABP5*).

### Functional annotation of genes regulated by eQTL

**Figure 4:**
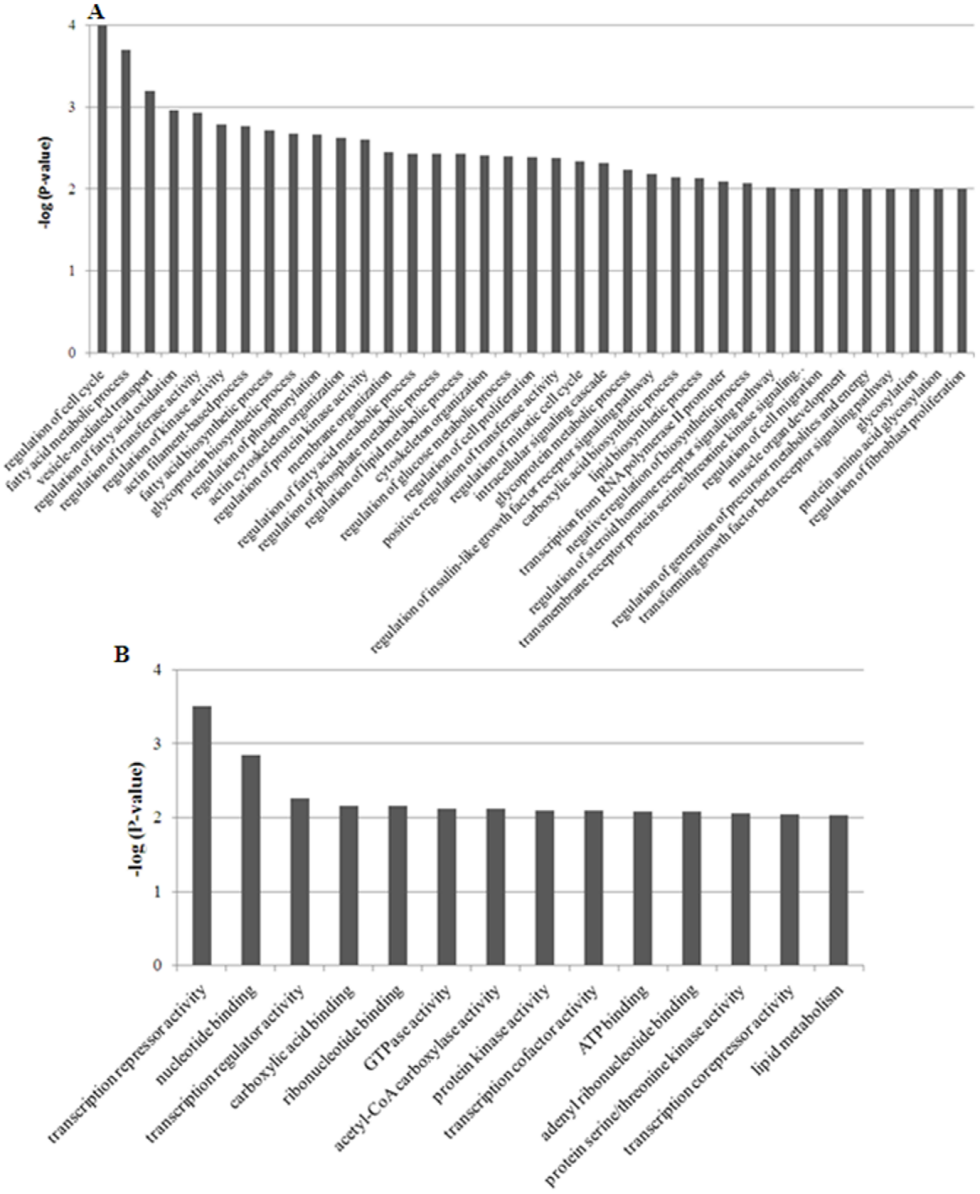
Global GO enrichment analysis performed with DAVID software on the list of eQTL-regulated genes. (A) List of most significant GO terms represented in the biological process category (*P<*0.01). (B) List of most significant GO terms represented in the molecular function category (*P<*0.01).

GO term annotations were used in order to perform a functional categorization of genes regulated by genome-wide significant eQTL (Figure 4). As a predictable consequence of our experimental design, which analysed two groups of pigs with divergent phenotypes for lipid traits, GO terms related with lipid metabolism were particularly abundant. In this context, the biological process GO category (Figure 4A) included *regulation of fatty acid oxidation, fatty acid biosynthetic process, regulation of fatty acid metabolic process*, *regulation of lipid metabolic process*, and *lipid biosynthetic process* among GO terms significantly enriched by our list of eQTL-regulated genes. Additionally, a variety of developmental and morphological processes related to muscle development and function (*e.g. actin filament-based process*, *cytoskeleton organization*, and *muscle organ development*) were also spread over the list of most significantly enriched GO terms. Other metabolic processes such as *regulation of glucose metabolic process* and *regulation of insulin-like growth factor receptor signalling* appeared in the middle of the list. Concerning the molecular function GO category (Figure 4B), *carboxylic acid binding*, *acetyl-CoA carboxylase activity* and *lipid metabolism* were among the most significantly enriched GO terms. The cellular component GO classification (data not shown) only indicated that most genes encoded intracellular components. Our findings partially agree with those obtained by Liaubet et al. [10], where most eQTL were engaged in functions related to muscle development and physiology, cell metabolism, cellular movement, cell-to-cell signalling and interaction and protein synthesis and posttranslational modification. Steibel et al. [9] also found that lipid metabolism, cell cycle, apoptosis and DNA replication, recombination and repair networks were particularly enriched in eQTL-regulated genes.

Within the general goal of investigating the segregation of muscle eQTL at the within-breed level, we were particularly interested on the genetic regulation of lipid metabolism and fatness traits. On the basis of results previously presented, eQTL-regulated genes potentially associated with muscle fat deposition and meat quality traits were further investigated. First of all, we performed a more refined analysis of the genes enriching the five lipid metabolism-related GO terms (Tables 3 and 4). These included eight *cis-* (*ABAT*, *ACER3, ANXA8L1*, *APOBEC1*, *EHD1*, *FIG4*, *PAFAH1B3* and *SNX14*) and 16 *trans-*regulated (*ACLY, ACACA*, *ACOT6*, *ARNTL*, *CAST*, *CS, CYP24A1*, *EHD1*, *FABP5*, *HRC, IGFBP5, IGF1, LRP6*, *PRKAA1*, *PRKAA* and *RRMB2*) loci. In general, *cis-*regulated genes were involved in either *(i)* modulation of intracellular levels of bioactive lipids that function as second messengers to cell and differentiation events, or *(ii)* lipid transport by lipoproteins. The list of 16 *trans-*regulated loci included well-studied genes related with fat deposition (*e.g. ACACA, FABP5, IGF1*, *ACLY* and *CS*) and meat tenderisation (*CAST*).

**Table 3:** Genome-wide significant *cis-*eQTL for *gluteus medius* expression traits whose target genes are functionally related to lipid metabolism and fat deposition.

	eQTL location and significance				QTL positional concordance in the same population
*Cis-*regulated Gene	Chromosome	Position	F-value	Nominal *P*-value	Linked Trait^1^
*SNX14*	SSC01	28 cM	4.81	0.000533	-
*FIG4*	SSC01	40 cM	5.10	0.000316	-
*NEDD4L*	SSC01	64 cM	5.03	0.000359	IMF
*EHD1*	SSC02	122 cM	6.16	0.000048	-
*ABAT*	SSC03	10 cM	5.62	0.000125	IMF; Vaccenic FA
*GSPT1*	SSC03	10 cM	5.60	0.000129	IMF; Vaccenic FA
*LCMT1*	SSC03	24 cM	7.78	0.000003	IMF
*MEPCE*	SSC03	24 cM	5.15	0.000289	IMF
*PPP1CB*	SSC03	24 cM	4.64	0.000725	IMF
*APOBEC1*	SSC05	124 cM	4.95	0.000414	-
*PAFAH1B3*	SSC06	70 cM	5.00	0.000379	Vaccenic FA
*IL12RB2*	SSC06	92 cM	5.13	0.000300	IMF
*SLA-1*	SSC07	72 cM	5.35	0.000202	Palmitoleic FA
*NUDT6*	SSC08	76 cM	6.94	0.000012	Myristic FA
*ACER3*	SSC09	138 cM	4.68	0.000675	-
*ANXA8L1*	SSC14	44 cM	4.78	0.000563	Palmitoleic FA
*ITGAV*	SSC15	68 cM	4.56	0.000838	MUFA; PUFA
*DNAJB6*	SSC18	40 cM	4.98	0.000393	Palmitic FA; SFA
*HOXA10*	SSC18	40 cM	4.68	0.000675	Palmitic FA; SFA

1 IMF – intramuscular fat content; FA – Fatty Acid content (%); SFA – % saturated fatty acids; MUFA – % monounsaturated fatty acids; PUFA – % polyunsaturated fatty acids.

**Table 4:** Genome-wide significant *trans-*eQTL for *gluteus medius* expression traits whose target genes are functionally related to lipid metabolism and fat deposition.

	eQTL location and significance					QTL positional concordance in the same population
*Trans-*regulated Gene	SSC	Position	F-value	Nominal *P*-value	Positional Genes^1^	Linked Trait^2^
*IGFBP5*	SSC01	10 cM	4.69	0.000663	*AKAP12*	-
*YWHAZ*	SSC01	68 cM	4.58	0.000808	*FBXL4*	IMF
*NFE2L1*	SSC02	92 cM	5.03	0.000359	*CAST*	Vaccenic FA
*ACOT6*	SSC02	122 cM	4.53	0.000885	*ACSL6*	-
*RRM2B*	SSC03	24 cM	4.58	0.000808	*XYLT1*	IMF
*NNAT*	SSC03	28 cM	5.44	0.000172	*LITAF*	IMF
*EHD1*	SSC03	34 cM	5.49	0.000157	*CREBBP*	-
*IGF1*	SSC03	58 cM	4.96	0.000407	*CTNNA2*	-
*PRKAA2*	SSC05	114 cM	5.26	0.000237	*ALG12*	-
*LRP6*	SSC06	66 cM	5.53	0.000147	*PPT1*	Vaccenic FA
*MTSS1*	SSC06	70 cM	5.11	0.000311	*LPIN2*	Vaccenic FA
*CS*	SSC06	94 cM	4.77	0.000573	*AK5*	IMF
*PRKAA1*	SSC06	144 cM	5.88	0.000079	*RNU6-1*	-
*FABP5*	SSC07	132 cM	4.64	0.000725	*PPP1R13B*	IMF
*ACACA*	SSC07	136 cM	4.85	0.000496	*ALPK3*	IMF
*ACLY*	SSC07	136 cM	4.53	0.000885	*ALPK3*	IMF
*CAST*	SSC08	74 cM	4.75	0.000594	*MGST2*	Myristic FA
*ARNTL*	SSC11	34 cM	5.22	0.000255	*KLHL1*	Muscle Cholesterol
*CYP24A1*	SSC18	38 cM	8.18	0.000001	*NPSR1*	Palmitic FA; SFA

1 Positional genes fitting in the confidence interval of the eQTL are indicated.

2 IMF – intramuscular fat content; FA – Fatty Acid content (%); SFA – % saturated fatty acids.

As a complementary approach, we performed a pathway analysis of the eQTL regulated genes. In clear consistency with the GO analysis, pathways involved in lipid metabolism and/or adipose/muscle tissue differentiation were amongst the most significant ones (Table 5). These included the *hypertrophic cardiomyopathy* and the *adipocytokine signalling* pathway on the top of the list. The *insulin signalling pathway*, associated with energy balance, was enriched by genes related to insulin resistance (*e.g. IRS1*) as well as by several genes involved in lipogenesis and cholesterogenesis (*e.g. ACACA*, *PRKAA1* and *PRKAA2*). Besides, the *PPAR signalling pathway* appeared in the middle of the list, gathering several genes with roles in lipid biosynthesis and fatty acid uptake, transport, and metabolism (*e.g. ACSL1*, *ACSL5* and *FABP5*). Finally, the *TGF-beta signalling* pathway, which regulates the switch of myocyte/adipocyte differentiation, was also present in this list.

**Table 5:** DAVID analysis of pathways significantly enriched in the list of *cis-* and *trans-*regulated genes.

KEGG id	KEGG pathway name	Genes	Genes (n)	*P*-value	Fold Enrichment
05410	**Hypertrophic cardiomyopathy (HCM)**	*LAMA2, ITGA6, ITGAV, PRKAG2, IGF1, PRKAA1, PRKAA2, TTN, TGFB2*	9	0.002	3.739
04920	**Adipocytokine signalling pathway**	*ACSL1, PRKAG2, PRKAA1, ACACB, PRKAA2, AGRP, IRS1, ACSL5*	8	0.003	4.216
04960	Aldosterone-regulated sodium reabsorption	*HSD11B1, IGF1, NEDD4L, ATP1A2, IRS1*	5	0.027	4.306
05219	Bladder cancer	*HRAS, MMP9, MDM2, CDK4, MMP1*	5	0.029	4.204
04144	Endocytosis	*STAMBP, HRAS, DNM1L, TGFBR1, RAB22A, SH3KBP1, ADRBK2, MDM2, NEDD4L, ZFYVE20, EHD1*	11	0.034	2.111
04910	**Insulin signalling pathway**	*HRAS, PPP1R3B, PRKAG2, ACACA, PRKAA1, ACACB, PRKAA2, PPP1CB, IRS1*	9	0.036	2.354
03320	**PPAR signalling pathway**	*ACSL1, SCD, MMP1, FABP5, ACOX3, ACSL5*	6	0.044	3.071
04510	Focal adhesion	*LAMA2, CAV2, HRAS, ITGA6, PDGFA, TNC, ITGAV, IGF1, COL1A1, PPP1CB, PARVB*	11	0.056	1.933
04110	Cell cycle	*YWHAZ, MAD2L1, CDC23, MDM2, CDK7, CDK4, CUL1, TGFB2*	8	0.061	2.260
05200	Pathways in cancer	*HRAS, PDGFA, MMP9, TGFBR1, IGF1, CDK4, MMP1, TGFB2, LAMA2, CCDC6, ITGA6, ITGAV, MDM2, TPR, GSTP1*	15	0.076	1.615
04350	**TGF-beta signalling pathway**	*TGFBR1, FST, ID4, BMPR1B, CUL1, TGFB2*	6	0.097	2.435

In bold, pathways related to lipid and muscle metabolism and fat deposition.

Taken as a whole, these findings suggest that genetic factors modulating muscle lipid metabolism exert their effects at multiple levels including lipid biosynthesis, absorption, transport and degradation. However, the specific polymorphisms involved in the genetic control of these processes may vary from population to population. In their experiment, closely paralleling ours, Steibel et al. [9] reported loin muscle eQTL in a divergent cross population that were mainly regulating three gene networks associated with lipid metabolism, DNA replication, and cell cycle regulation. The high consistency between these two experiments suggests that, in spite of the fact that gene pools are different, the regulatory mechanisms that modify the porcine skeletal muscle transcriptome are very similar across pig breeds.

Finally, it is worth mentioning that 306 (41 *cis-* and 267 *trans-*eQTL) out of 455 eQTL-regulated loci matched to genes that happened to be differentially expressed between pigs displaying divergent fatness profiles [11]. Amongst these genes, we identified loci related with fat deposition (*e.g. ACACA*, *ACSL1*, *ACSL5*, *ACLY*, *FABP5*, *CEBPD*, *IGFBP5* and *SCD*) that showed at least one *trans-*regulatory polymorphism. This result suggests that the divergent fatness phenotypes characterized in our purebred Duroc population by Canovas et al. [11] might have, in part, a genetic origin *i.e.* they might be due to the differential segregation (in the High and Low groups) of regulatory polymorphisms with effects on lipid metabolism-related genes. Further research dealing with differential allelic segregation analyses at a whole-genome level would shed light on this topic.

### Positional concordance between eQTL and QTL for lipid metabolism and fat deposition traits

Combining eQTL and QTL linkage maps represents an interesting approach to identifying polymorphisms regulating phenotypic variation through changes in gene expression. We have carried out a positional concordance study between 455 eQTL (with a high quality mapped target probes) and a dataset of QTL for IMF, muscle cholesterol content and fatty acid profile segregating in the same Duroc population [14] (Tables 3 and 4). In this way, we identified 80 positional concordances, which corresponded to 14 *cis-*acting and 66 *trans-*acting eQTL. We were particularly interested in those eQTL-QTL pairs involving genes functionally related with lipid metabolism and meat quality. In this regard, as much as five IMF QTL showed positional concordance with eQTL regulating lipid metabolism- and adipose function-related genes. It is particularly remarkable the co-localisation of a highly significant IMF QTL at SSC7 and a *trans-*acting regulatory hotspot influencing, among others, the expression levels of *ACACA*, *ACLY* and *FABP5* mRNA. These three genes have a well-known role in fat metabolism and showed a differential mRNA expression pattern in pigs with divergent phenotypes for fatness traits [11]. All these evidences point out this SSC7 region (Figure 3) as a major genetic factor modifying lipid traits, as previously demonstrated in several QTL studies [14], [23], [24], [25]. Similarly, we have identified a SSC3 region harbouring a highly significant IMF QTL [14], [16], [26] and an eQTL hotspot (Figure 3). This *trans-*regulatory hotspot is associated with at least seven genes (Table S1) including *RRM2B*, which plays a relevant role in the processes of fatty acid β-oxidation and lipid synthesis [27] (Table 4).

QTL affecting muscle fatty acid composition (such as vaccenic, myristic and palmitic fatty acids percentage) at SSC3, SSC6, SSC8, SSC14 and SSC18 also co-localised with several *cis-*and *trans-*acting eQTL (Table 3 and 4). Amongst the genes regulated by these eQTL it is worth mentioning *LRP6*, which encodes a co-receptor for Wnt factors and LDL lipoproteins. This molecule is involved in lipid uptake by adipocytes through regulation of early adipogenesis events [28] and receptor-mediated endocytosis of LDL lipoproteins [29]. Besides, it is worth to mention a QTL for muscle cholesterol content at SSC11 that co-localised with an eQTL affecting the expression of *ARNTL* (Table 4). *ARNTL* is a clock gene involved in the circadian rhythm of cholesterol (and other lipids) accumulation, having been linked to susceptibility to the metabolic syndrome in humans [30].

The existence of eQTL-QTL positional concordance may be interpreted as evidence that a number of regulatory polymorphism, and more particularly those located at eQTL-hotspot regions on SSC3 and SSC7, display relevant associations with phenotypes of economic interest (*i.e.* IMF content and composition traits), probably through the regulation of a number of genes related with lipid metabolism and other biological functions (Table 3 and 4). Conceivably, polymorphisms with quantitative effects on the transcriptome might be also partially responsible for phenotypic variability of IMF content and composition. Under this assumption, studies combining eQTL-QTL scans, optimally in combination with gene network/biological systems approaches, should be considered as the most powerful strategy to identify causal genes and mutations. It is fundamental to stress, however, that finding overlaps between eQTL and QTL, although suggestive from a biological point of view, does not allow establishing a causal connection [31]. In fact, correlations due to linkage disequilibrium and/or fortuitous overlaps are expected to occur because of the low resolution of microsatellite QTL mapping (QTL intervals are large) and the high density of eQTL maps (they cover a substantial part of the genome).

### Correlation analysis between target gene expression and muscle lipid phenotypes

A correlation study was performed in order to confirm the relationship between mRNA expression of certain genes and phenotypic variation. A total of 16 (*ACACA*, *ACLY*, *ARNTL*, *CAST*, *CS*, *CYP24A1*, *DNAJB6*, *FABP5*, *HOXA10*, *IL12RB2*, *LRP6*, *NEDD4L*, *NUDT6*, *PPP1CB*, *RRM2B* and *YWHAZ*) out of 80 genes that were regulated by eQTL mapping to the confidence interval of QTL were selected for the correlation study, on the basis of their functional relationship with muscle fat deposition. We computed the correlation coefficients between their mRNA expression levels and muscle fat deposition traits, including IMF, muscle cholesterol content and fatty acid composition. Correlation coefficients that were significant in at least one of the three calculations (whole dataset or within High or Low fatness groups) are shown in Table 6. As a whole, correlation coefficients between gene expression levels and IMF content and composition phenotypes were moderate (positive and negative), displaying absolute values that ranged from 0.25 to 0.44 (Table 6). A few robust and positive correlations were observed between *NEDD4L* mRNA levels and myristic fatty acid content, and between *CYP24A1* expression levels and palmitic fatty acid content. However, consistency between correlation coefficients obtained within the two groups of pigs with divergent profiles for lipid traits was, in general, quite low. For instance, the mRNA levels of the *ACACA* and *ACLY* genes showed positive correlation coefficients with IMF content, but this relationship did not reach statistical significance in the High group. Similarly, muscle gene expression of *IL12RB2* was positively correlated with IMF content but only in the High group (Table 6). Even more, opposite relationships (negative correlation in one group, positive in the other one) were observed when comparing the High and Low groups (*e.g.* correlation of *CS*, *CAST*, *LRP6* and *RRM2B* mRNA levels with linoleic fatty acid content in muscle). Opposite correlation relationship in extreme groups had been reported before between *SCD* protein expression levels and oleic acid muscle composition [32], probably as an effect of selection for favourable alleles.

**Table 6:** Correlation coefficients (r) between muscle mRNA expression of several *cis-* and *trans-* regulated genes and intramuscular fat content, fatty acid profile and cholesterol content of the *gluteus medius* muscle.

			High group^2^		Low group^2^		Whole dataset^2^	
	Regulated gene	QTL trait^1^	R	*P*-value	r	*P*-value	r	*P*-value
	***DNAJB6***	Linoleic FA	−0.08	0.5729	**−0.30**	**0.0339**	−0.18	0.0670
	***HOXA10***	Palmitoleic FA	**0.39**	**0.0037**	0.01	0.9643	0.19	0.0526
		Linoleic FA	**0.35**	**0.0100**	−0.22	0.1250	0.08	0.4191
		Stearic FA	**−0.32**	**0.0227**	−0.09	0.5429	**−0.21**	**0.0388**
***cis-*** **acting**	***IL12RB2***	IMF	**0.37**	**0.0062**	0.16	0.2565	**0.32**	**0.0008**
**eQTL**		Muscle Cholesterol	0.16	0.2377	**−0.31**	**0.0261**	0.13	0.1713
	***NEDD4L***	Myristic FA	**0.28**	**0.0367**	**0.30**	**0.0310**	**0.33**	**0.0006**
		Palmitoleic FA	**0.28**	**0.0425**	−0.04	0.7929	−0.03	0.7984
	***NUDT6***	Palmitic FA	0.04	0.7829	**−0.33**	**0.0293**	−0.14	0.1602
	***PPP1CB***	Palmitic FA	**0.29**	**0.0380**	−0.13	0.4082	0.14	0.1712
		Linoleic FA	−0.22	0.1035	**0.36**	**0.0099**	−0.02	0.8783
	***ACACA***	IMF	0.15	0.2669	**0.43**	**0.0016**	**0.42**	**0.0001**
	***ACLY***	IMF	−0.02	0.8688	**0.31**	**0.0248**	0.18	0.0623
		Palmitic FA	0.04	0.7942	**0.37**	**0.0123**	**0.23**	**0.0264**
	***CAST***	Palmitoleic FA	**−0.32**	**0.0189**	−0.25	0.0713	**−0.32**	**0.0010**
		Myristic FA	0.25	0.0708	**−0.29**	**0.0381**	0.09	0.3623
		Linoleic FA	−0.23	0.0832	**0.35**	**0.0107**	−0.06	0.5432
		Palmitic FA	**0.31**	**0.0297**	−0.04	0.7828	**0.22**	**0.0310**
	***CS***	Palmitoleic FA	**0.29**	**0.0318**	−0.03	0.8497	0.15	0.1237
		Linoleic FA	**0.39**	**0.0039**	**−0.33**	**0.0188**	0.07	0.4947
	***CYP24A1***	Palmitic FA	**0.25**	**0.0468**	**0.38**	**0.0108**	**0.26**	**0.0100**
***trans-*** **acting**		Muscle Cholesterol	**−0.32**	**0.0172**	**−0.34**	**0.0134**	−0.17	0.0890
**eQTL**	***FABP5***	Palmitoleic FA	**−0.41**	**0.0023**	0.18	0.2045	0.05	0.6454
		Linoleic FA	**−0.35**	**0.0086**	0.12	0.4153	0.07	0.4594
		Stearic FA	**0.44**	**0.0014**	0.01	0.9986	0.15	0.1449
		Muscle Cholesterol	**−0.27**	**0.0453**	0.05	0.7523	−0.12	0.2088
	***LRP6***	Palmitoleic FA	**0.27**	**0.0444**	0.08	0.5585	0.08	0.4374
		Myristic FA	0.01	0.9834	**0.31**	**0.0286**	0.16	0.1023
		Linoleic FA	0.24	0.0859	**−0.41**	**0.0027**	−0.15	0.1392
	***RRM2B***	Linoleic FA	**−0.29**	**0.0312**	**0.40**	**0.0032**	0.04	0.6706
		Stearic FA	**0.29**	**0.0385**	0.05	0.7690	0.18	0.0755
	***YWHAZ***	Palmitoleic FA	**0.33**	**0.0148**	0.21	0.1494	**0.28**	**0.0040**
		Linoleic FA	**0.33**	**0.0151**	−0.17	0.2205	0.11	0.2434

1 IMF – intramuscular fat content; FA – Fatty Acid content (%).

2 Significant correlation coefficients are indicated in bold.

Taken as a whole, this correlation study provided limited evidences of a linear relationship between muscle fat deposition and mRNA levels of genes functionally related with lipid metabolism. Discrepancies between correlations estimated in the two groups might be explained by experimental (*i.e.* limited sample size) and biological (*i.e.* gene expression might be modulated to some extent by the metabolic profile of individuals) factors. In this sense, it is well known that obesity and leanness, as “metabolic states”, can influence the expression of many genes, including those that are completely unrelated with lipid metabolism [11], [33]. For instance, the increased expression of proinflammatory cytokines by the adipose tissue of obese individuals is explained by its substantial infiltration by macrophages [34]. In this same line, Cánovas et al. [11] also showed important differences in the expression of cell differentiation, energy balance and fat metabolism genes between pigs with divergent lipid metabolic profiles. Finally, the possibility of epistatic interactions, jointly with the differential segregation of regulatory polymorphisms, would be also worth to be explored as a potential cause of divergent expression-phenotype relationships between High and Low groups. In any case, it is necessary to emphasize that correlations should not be interpreted in terms of causality, but as a measure of the association between two variables. In other words, the existence of a correlation between gene expression and phenotypic variation of complex traits is suggestive from a biological point of view, but it does not formally proof the existence of a functional link between both datasets.

## Materials and Methods

### Animal material and microsatellite genotyping

An experimental pig population (350 castrated males) distributed in five half-sib families and four contemporary batches was generated from a commercial Duroc line mainly devoted to the production of fine quality dry-cured products. This commercial line was selected to increase intramuscular fat (IMF) content, jointly with prolificacy and growth traits, while maintaining carcass fatness. Inbreeding was controlled by an appropriate mating design and the sporadic introgression of genetic material. Pigs were castrated and controlled at IRTA-Pig Control Centre facilities. A number of phenotypes related with fatness, serum lipid levels, and IMF content and composition were recorded and measured as previously described by Gallardo et al. [13], [35]. So far, several investigations including QTL mapping for lipid metabolism and fat deposition traits [13], [14], [15] and a differential mRNA expression study [11] have been conducted in this commercial population. The experimental procedures, phenotype recording and blood sampling were approved by the Ethical Committee of the Institution (IRTA- *Institut de Recerca i Tecnologia Agroalimentàries*).

The Duroc commercial population was genotyped for 116 informative microsatellites covering the 18 autosomes with an average interval space between markers of 19.2 cM. All the information related to microsatellite genotyping and the description of the linkage map can be found in Quintanilla et al. [14].

### Expression data and normalization

We have analysed the global mRNA expression profile of *gluteus medius* muscle samples obtained from 105 Duroc pigs distributed in two groups with divergent phenotypes for fatness traits (53 and 52 individuals in the High and Low groups, respectively). Groups were established on the basis of multivariant analyses for several lipid deposition traits, as described in Cánovas et al. [11]. RNA isolation and hybridization in *GeneChip Porcine Genome®* arrays (Affymetrix, Inc., Santa Clara, CA) procedures are also reported in Cánovas et al. [11]. Microarray data normalisation was assessed through the gcRMA algorithm, which corrects the intensity of each probe by its GC content [36], using the BRB-ArrayTools software version 3.7.1 [37], which is available online at http://linus.nci.nih.gov/BRB-ArrayTools.html. Microarray data obtained in the current study have been deposited in the Gene Expression Omnibus (GEO) public repository with GSE19275 and GSE26091 GEO accession numbers.

Before performing the eQTL scan, the “minimum fold-change” filter implemented in the BRB-ArrayTools was applied in order to filter out those probes displaying low expression variability across the 105 muscle samples. The criterion consisted on selecting those probes with more than 20% of its expression values (from the 105 arrays) meeting at least ±1.5 times the median expression of the probe.

### Expression quantitative trait loci analyses

A genome-wide eQTL scan was carried out for 6,139 *Affymetrix* probes which had passed the “minimum fold-change” filter described above. These eQTL analyses were performed for each probe, using the aforementioned panel of 116 microsatellites, by means of the *GridQTL* software [38], available at http://www.gridqtl.org.uk/. The common model used for eQTL scanning was:
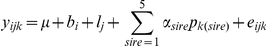
where: *y_ijk_* is the expression data of individual *k*; *b_i_* is the effect of *i^th^* contemporary batch of fattening (four levels); *l_j_* is the laboratory *j* effect (two levels); *α* is the regression coefficient (mean allele substitution effect); *p_k_* is the probability of individual *k* inheriting a given allele from its common parent; *e_ijk_* is the residual effect. Genome-wide significance thresholds for the F-values (eQTL model *vs* no eQTL model) were approximated with the Bonferroni correction as described in Gallardo et al. [13]. Genome-wide significance levels of 95% and 99% corresponded to *P*-values of 0.0009 and 0.0002 respectively.

### Reference assembly and mapping analyses

For those target probes regulated by significant eQTL, we analysed the *Affymetrix* porcine probe-sets (11 probes per probe-set). These probes were assembled to the pig genome sequence with the Ensembl annotation tool (http://www.ensembl.org/info/data/ftp/index.html) using CLCBio workbench software (CLC Bio, Aarhus, Denmark), and quality of all significant probes was thoroughly examined. In the mapping analysis, we took into account the proportion of mismatches with the reference sequence. In this way, probes were classified into uniquely mapped probes and non-specifically mapped probes.

The GBrowse tool, available at the pig QTL database (http://www.animalgenome.org/cgi-bin/gbrowse/pig/), was used to locate all probes affected by significant eQTL as well as to align these eQTL to the pig genome. The alignment of eQTL genomic locations against target transcripts or genes was accomplished by converting the linkage eQTL map (cM) to genome sequence assembly locations (bp) as described in Hu et al. [39]. This was achieved by taking as a reference anchoring markers that are mapped on both (linkage and genome) maps. Whenever eQTL boundaries did not coincide with anchoring marker locations, relative genome locations were calculated with an algorithm that takes into account the chromosomal length, the cM *vs* bp ratio, and the offset of the eQTL location to that of anchoring marker [40]. The comparison between eQTL and their corresponding target gene locations allowed us to classify eQTL in *cis-*acting eQTL (close to the target gene) and *trans-*acting eQTL (in different chromosome or far away from the target gene). As in Ponsuksili et al. [16], *cis-*acting eQTL were defined as those in which the target gene position falls within the interval delimited by the two flanking markers of the eQTL peak.

### Functional annotation: Gene Ontology and Pathway analyses

Gene Ontology (GO) describes the basic hierarchies and relationships between terms within the context of biology. The three GO categories (biological process, molecular function and cellular component) were analysed in order to achieve a full coverage of the GO spectrum. The GO term enrichment analyses for overrepresentation of specific GO terms were carried out with the Database for Annotation, Visualization and Integrated Discovery (DAVID) bioinformatic package, available at http://david.abcc.ncifcrf.gov. Significance levels were computed following a modification of Fisher's exact test. Multiple testing-corrected *P*-values were also obtained using the Benjamini and Hochberg algorithm, and only GO terms with Benjamini-corrected *P*-values <0.01 were considered. DAVID was additionally used to explore the biological pathways enriched in the resulting eQTL-regulated gene list, computing a total overrepresentation value for each pathway represented in the Kyoto Encyclopaedia of Genes and Genomes (KEGG, http://www.genome.jp/kegg).

### Correlation analyses between gene expression levels and muscle lipid phenotypes

Correlation analyses between expression levels and several phenotypes related to lipid metabolism and fat deposition were additionally carried out for a number of eQTL-regulated genes selected on the basis of the former analyses. The correlation study was performed using the CORR procedure of SAS (SAS Institute Inc., Cary, NC) after adjusting phenotypes and expression levels for the environmental significant effects considered in previous analyses (residual correlations). Two correlations analyses were carried out: 1) correlation analyses considering the whole dataset (n = 105 for each correlation); and 2) within-group correlation analyses, considering separately the two groups of individuals with divergent lipid metabolism profiles (n = 53 and 52 for the High and Low groups, respectively).

## Supporting Information

Table S1 **Genome-wide significant eQTL with high quality target probes: eQTL peaks, target gene locations, flanking markers, confidence intervals, distances from the closest marker, and classification as** ***cis*** **-/** ***trans*** **- eQTL.**
(XLS)

## References

[1] Emilsson V, Thorleifsson G, Zhang B, Leonardson AS, Zink F (2008). Genetics of gene expression and its effect on disease.. Nature.

[2] Schadt EE, Molony C, Chudin E, Hao K, Yang X (2008). Mapping the genetic architecture of gene expression in human liver.. PLoSBiol.

[3] Zhang W, Duan S, Kistner EO, Bleibel WK, Huang RS (2008). Evaluation of genetic variation contributing to differences in gene expression between populations.. Am J Hum Genet.

[4] Duan S, Huang RS, Zhang W, Bleibel WK, Roe CA (2008). Genetic architecture of transcript-level variation in humans.. Am J Hum Genet.

[5] Cheung VG, Spielman, RS (2009). Genetics of human gene expression: mapping DNA variants that influence gene expression.. Nature Reviews Genetics.

[6] Gilad Y, Rifkin SA, Pritchard JK (2008). Revealing the architecture of gene regulation: the promise of eQTL studies.. Trends Genet.

[7] Wimmers K, Murani E, Ponsuksili S (2010). Functional genomics and genetical genomics approaches towards elucidating networks of genes affecting meat performance in pigs.. Brief Funct Genomics.

[8] Ponsuksili S, Murani E, Brand B, Schwerin M, Wimmers K (2011). Integrating expression profiling and whole-genome association for dissection of fat traits in a porcine model.. J Lipid Res.

[9] Steibel JP, Bates RO, Rosa GJ, Tempelman RJ, Rilington VD (2011). Genome-wide linkage analysis of global gene expression in loin muscle tissue identifies candidate genes in pigs.. PLoS One.

[10] Liaubet L, Lobjois V, Faraut T, Tircazes A, Benne F (2011). Genetic variability of transcript abundance in pig peri-mortem skeletal muscle: eQTL localized genes involved in stress response, cell death, muscle disorders and metabolism.. BMC Genomics.

[11] Cánovas A, Quintanilla R, Amills M, Pena RN (2010). Muscle transcriptomic profiles in pigs with divergent phenotypes for fatness traits.. BMC Genomics.

[12] Casellas J, Noguera JL, Reixach J, Díaz I, Amills M, Quintanilla R (2010). Bayes factor analyses of heritability for serum and muscle lipid traits in Duroc pigs.. J Anim Sci.

[13] Gallardo D, Pena RN, Amills M, Varona L, Ramírez O (2008). Mapping of quantitative trait loci for cholesterol, LDL, HDL and triglyceride serum concentrations in pigs.. Physiol Genomics.

[14] Quintanilla R, Pena RN, Gallardo D, Cánovas A, Ramírez O (2011). Porcine intramuscular fat content and composition are regulated by quantitative trait loci with muscle-specific effects.. J Anim Sci.

[15] Gallardo D, Pena RN, Quintanilla R, Ramírez O, Almuzara D (2012). Quantitative trait loci analysis of a Duroc commercial population highlights differences in the genetic determination of meat quality traits at two different muscles.. Anim Genet..

[16] Ponsuksili S, Jonas E, Murani E, Phatsara C, Srikanchai T (2008). Trait correlated expression combined with expression QTL analysis reveals biological pathways and candidate genes affecting water holding capacity of muscle.. BMC Genomics.

[17] Göring HH, Curran CJ, Johnson MP, Dyer TD, Charlesworth J (2007). Discovery of expression QTLs using large-scale transcriptional profiling in human lymphocytes.. Nat Genet.

[18] Morley M, Molony CM, Weber TM, Devlin JL, Ewens KG (2004). Genetic analysis of genome-wide variation in human gene expression.. Nature.

[19] Myers AJ, Gibbs GJ, Webster JA, Rohrer K, Zhao A (2007). A survey of genetic human cortical gene expression.. Nature Genet.

[20] Cheung VG, Nayak RR, Wang IX, Elwyn S, Cousins SM (2010). Polymorphic cis- and trans-regulation of human gene expression.. PLoS Biol.

[21] Yvert G, Brem RB, Whittle J, Akey JM, Foss E (2003). Trans-acting regulatory variation in Saccharomyces cerevisiae and the role of transcription factors.. Nat Genet.

[22] Hubner N, Wallace CA, Zimdahl H, Petretto E, Schulz H (2005). Integrated transcriptional profiling and linkage analysis for identification of genes underlying disease.. Nat Genet.

[23] de Koning DJ, Janss LL, Rattink AP, van Oers PA, de Vries BJ (1999). Detection of quantitative trait loci for backfat thickness and intramuscular fat content in pigs (Sus scrofa).. Genetics.

[24] Nagamine Y, Haley CS, Sewalem A, Visscher PM (2003). Quantitative trait loci variation for growth and obesity between and within lines of pigs (Sus scrofa).. Genetics.

[25] Sato S, Hasebe H, Sato S, Asahi Y, Hayashi T (2006). High-resolution physical mapping and construction of a porcine contig spanning the intramuscular fat content QTL.. Anim Genet.

[26] de Koning DJ, Pong-Wong R, Varona L, Evans GJ, Giuffra E (2003). Full pedigree quantitative trait locus analysis in commercial pigs using variance components.. J Anim Sci.

[27] Huang CC, Hsu CH (2009). Mitochondrial disease and mitochondrial DNA depletion syndromes.. Acta Neurol Taiwan.

[28] Christdoulides C, Laudes M, Cawthorn WP, Schinner S, Soos M (2006). The Wnt antagonist Dickkopf-1 and its receptors are coordinately regulated during early human adipogenesis.. J Cell Sci.

[29] Tomaszewski M, Charchar FJ, Barnes T, Gawron-Kiszka M, Sedkowska A (2009). A common variant in low-density lipoprotein receptor-related protein 6 gene (LRP6) is associated with LDL-cholesterol.. Arterioscler Thromb Vasc Biol.

[30] Gómez-Abellán P, Hernández-Morante JJ, Luján JA, Madrid JA, Garaulet M (2008). Clock genes are implicated in the human metabolic syndrome.. Int J Obes.

[31] George M (2007). Mapping, Fine Mapping, and Molecular Dissection of Quantitative Trait Loci in Domestic Animals.. Ann Rev Genomics & Human Genetics.

[32] Canovas A, Estany J, Tor M, Pena RN, Doran O (2009). Acetyl-CoA carboxylase and stearoyl-CoA desaturase protein expression in subcutaneous adipose tissue is reduced in pigs selected for decreased backfat thickness at constant intramuscular fat content.. J Anim Sci.

[33] Balistreri CR, Caruso C, Candore G (2010). The role of adipose tissue and adipokines in obesity-related inflammatory diseases.. Mediators Inflamm.

[34] Morris DL, Singer K, Lumeng CN (2011). Adipose tissue macrophages: phenotypic plasticity and diversity in lean and obese states.. Curr Opin Clin Nutr Metab Care.

[35] Gallardo D, Quintanilla R, Varona L, Diaz I, Ramirez O (2009). Polymorphism of the pig acetyl-coenzyme A carboxylase alpha gene is associated with fatty acid composition in a Duroc commercial line.. Anim Genet.

[36] Wu Z, Airizarry R, Gentleman R, Martinez-Murillo F, Spencer F (2004). A Model-Based Background Adjustment for Oligonucleotide Expression Arrays.. J Amer Statist Asso.

[37] Xu X, Zhao Y, Simon R (2008). Gene Set Expression Comparison kit for BRB-ArrayTools.. Bioinformatics.

[38] Seaton G, Hernandez J, Grunchec JA, White I, Allen J (2006). GridQTL: A grid portal for QTL mapping of compute intensive datasets.. In *Proc 8th WCGALP*.

[39] Hu ZL, Fritz ER, Reecy JM (2007). Animal QTLdb: a livestock QTL database tool set for positional QTL information mining and beyond.. Nuc Acid Res.

[40] Hu ZL, Reecy JM (2007). Animal QTLdb: beyond a repository. A public platform for QTL comparisons and integration with diverse types of structural genomic information.. Mamm Genome.

